# Physiological responses during ascent to high altitude and the incidence of acute mountain sickness

**DOI:** 10.14814/phy2.14809

**Published:** 2021-04-27

**Authors:** Alexandra B. Cobb, Denny Z. H. Levett, Kay Mitchell, Wynne Aveling, Daniel Hurlbut, Edward Gilbert‐Kawai, Philip J. Hennis, Monty G. Mythen, Michael P. W. Grocott, Daniel S. Martin, Caudwell Xtreme Everest, Xtreme Everest 2009, Xtreme Everest 2 investigators, V Ahuja, G Aref‐Adib, R Burnham, A Chisholm, K Clarke, D Coates, M Coates, D Cook, M Cox, S Dhillon, C Dougall, P Doyle, P Duncan, M Edsell, L Edwards, L Evans, P Gardiner, M Grocott, P Gunning, N Hart, J Harrington, J Harvey, C Holloway, D Howard, D Hurlbut, C Imray, C Ince, J van der Kaaij, M Khosravi, N Kolfschoten, D Levett, H Luery, A Luks, D Martin, R McMorrow, P Meale, K Mitchell, H Montgomery, G Morgan, J Morgan, A Murray, M Mythen, S. Newman, M O’Dwyer, J Pate, T Plant, M Pun, P Richards, A Richardson, G Rodway, J Simpson, C Stroud, M Stroud, J Stygal, B Symons, P Szawarski, A Van Tulleken, C Van Tulleken, A Vercueil, L Wandrag, M Wilson, J Windsor, B Basnyat, C Clarke, T Hornbein, J Milledge, J West, S Abraham, T Adams, W Anseeuw, R Astin, B Basnyat, O Burdall, J Carroll, A Cobb, J Coppel, O Couppis, J Court, A Cumpstey, T Davies, S Dhillon, N Diamond, C Dougall, T Geliot, E Gilbert‐Kawai, G Gilbert‐Kawai, E Gnaiger, M Grocott, C Haldane, P Hennis, J Horscroft, D Howard, S Jack, B Jarvis, W Jenner, G Jones, J van der Kaaij, J Kenth, A Kotwica, R Kumar BC, J Lacey, V Laner, D Levett, D Martin, P Meale, K Mitchell, Z Mahomed, J Moonie, A Murray, M Mythen, P Mythen, K O’Brien, I Ruggles‐Brice, K Salmon, A Sheperdigian, T Smedley, B Symons, C Tomlinson, A Vercueil, L Wandrag, S Ward, A Wight, C Wilkinson, S Wythe, M Feelisch, E Gilbert‐Kawai, M Grocott, M Hanson, D Levett, D Martin, K Mitchell, H Montgomery, R Moon, A Murray, M Mythen, M Peters

**Affiliations:** ^1^ University College London Centre for Altitude Space and Extreme Environment Medicine UCLH NIHR Biomedical Research Centre Institute of Sport and Exercise Health London UK; ^2^ Anaesthesia and Critical Care Research Unit University Hospital Southampton NHS Foundation Trust Southampton UK; ^3^ Perioperative and Critical Care Research Theme NIHR Biomedical Research Centre, University Hospital Southampton NHS Foundation Trust Southampton UK; ^4^ Integrative Physiology and Critical Illness Group, School of Clinical and Experimental Sciences, Faculty of Medicine University of Southampton Southampton UK; ^5^ Anaesthetic Department University College London Hospital London UK; ^6^ Intensive Care Unit University Hospitals Plymouth Plymouth UK; ^7^ Peninsula Medical School University of Plymouth Plymouth UK

**Keywords:** altitude, altitude sickness, exercise, hypoxia

## Abstract

Acute mountain sickness (AMS) occurs when there is failure of acclimatisation to high altitude. The aim of this study was to describe the relationship between physiological variables and the incidence of AMS during ascent to 5300 m. A total of 332 lowland‐dwelling volunteers followed an identical ascent profile on staggered treks. Self‐reported symptoms of AMS were recorded daily using the Lake Louise score (mild 3–4; moderate‐severe ≥5), alongside measurements of physiological variables (heart rate, respiratory rate (RR), peripheral oxygen saturation (SpO_2_) and blood pressure) before and after a standardised Xtreme Everest Step‐Test (XEST). The overall occurrence of AMS among participants was 73.5% (23.2% mild, 50.3% moderate–severe). There was no difference in gender, age, previous AMS, weight or body mass index between participants who developed AMS and those who did not. Participants who had not previously ascended >5000 m were more likely to get moderate‐to‐severe AMS. Participants who suffered moderate‐to‐severe AMS had a lower resting SpO_2_ at 3500 m (88.5 vs. 89.6%, *p* = 0.02), while participants who suffered mild or moderate‐to‐severe AMS had a lower end‐exercise SpO_2_ at 3500 m (82.2 vs. 83.8%, *p* = 0.027; 81.5 vs. 83.8%, *p* < 0.001 respectively). Participants who experienced mild AMS had lower end‐exercise RR at 3500 m (19.2 vs. 21.3, *p* = 0.017). In a multi‐variable regression model, only lower end‐exercise SpO_2_ (OR 0.870, *p* < 0.001) and no previous exposure to altitude >5000 m (OR 2.740, *p*‐value 0.003) predicted the development of moderate‐to‐severe AMS. The Xtreme Everest Step‐Test offers a simple, reproducible field test to help predict AMS, albeit with relatively limited predictive precision.

## Graphical abstract

We evaluated the performance of a simple exercise challenge at altitude (the Xtreme Everest Step‐Test) in predicting acute mountain sickness (AMS) on a trek to Everest Base Camp (5300 m). The overall occurrence of AMS during the trek was high (73.5%). Lower peripheral oxygen saturation (SpO_2_) following the Xtreme Everest Step‐Test at 3500 m and no previous exposure to altitude >5000 m predicted the development of moderate to severe AMS (Lake Louise score ≥5) during the trek. The Xtreme Everest Step‐Test offers a simple, reproducible field test to contribute towards the prediction of AMS, albeit with relatively limited predictive precision.

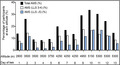

**Key points**

- We evaluated the performance of a simple exercise challenge at altitude (the Xtreme Everest Step‐Test) in predicting acute mountain sickness (AMS) on a trek to Everest Base Camp (5300 m).
- The overall occurrence of AMS during the trek (using the 1993 Lake Louise score threshold of ≥3) was high (73.5%).
- Participant characteristics previously shown to predict AMS (gender, age, weight and history of AMS) were not related to the development of AMS in this study.
- Lower peripheral oxygen saturation (SpO_2_) following the Xtreme Everest Step‐Test at 3500 m and no previous exposure to altitude >5000 m predicted the development of moderate‐to‐severe AMS (1993 Lake Louise score threshold of ≥5) during the trek.
- The Xtreme Everest Step‐Test offers a simple, reproducible field test to contribute towards the prediction of AMS, albeit with relatively limited predictive precision.

## 1 INTRODUCTION

Prior to the COVID‐19 pandemic, increasing numbers of people were travelling to high altitude, and it is likely that these numbers will return when domestic and international travel is freely available once again. During ascent to altitude, exposure to hypobaric hypoxia due to the reduction in partial pressure of oxygen occurs with increasing elevation. Acclimatisation permits adaptation to hypoxia, but failure of this process can result in acute mountain sickness (AMS). AMS is a syndrome that is defined by the presence of a headache in combination with other symptoms including dizziness, fatigue, loss of appetite and insomnia. AMS is relatively common among trekkers, affecting 36.7% of participants at 3658 m and as many as 75% of participants attempting Mount Kilimanjaro (5984 m; Gonggalanzi et al., 2016; Karinen et al., 2008). The Lake Louise scoring system was devised to aid in the diagnosis of AMS, provide a subjective scale for the description of symptoms and to facilitate research (Roach et al., 1993). The system was revised in 2018, by the removal of one scoring domain (Roach, 2018). The precise pathophysiology underlying AMS remains elusive, however, a number of hypotheses exist that may explain why the hypobaric hypoxia experienced at high altitude causes these symptoms (Wilson et al., 2009). SEVERAL risk factors for susceptibility to AMS have been previously identified, although published data have been conflicting; these include a history of previous AMS, younger age, female gender, rapid ascent, obesity and increased exertion (Hackett & Roach, 2001; Honigman et al., 1993; MacInnis et al., 2013; Richalet et al., 2012).

Results from studies attempting to predict which individuals will suffer from AMS in simulated altitude based on objective physiological measures have been inconsistent (Burtscher et al., 2008). The largest prospective cohort study to assess physiological risk factors for AMS using a hypoxic pre‐travel exercise test showed that high delta peripheral oxygen saturation (SpO_2_; beginning to end of exercise) and low ventilatory response to exercise were independent predictors of severe high‐altitude illness (Richalet et al., 2012). Additional risk factors identified were ascent >400 m per day, previous history of severe high‐altitude illness and previous history of migraine. Other studies assessing the use of resting SpO_2_ measurements to predict AMS at real or simulated altitude have been conflicting with some studies reporting an association between these two measures (Burtscher et al., 2004; Faulhaber et al., 2014; Guo et al., 2014; Karinen et al., 2010; Mandolesi et al., 2014), while other studies have not (Chen et al., 2012; Leichtfried et al., 2015; O’Connor et al., 2004; Wagner et al., 2012).

The aim of the current study was to describe, in a large cohort of healthy lowland‐dwelling volunteers, the relationship between simple physiological variables, measured before and after a standardised exercise protocol and the incidence of AMS during ascent to 5300 m following an identical ascent profile. We hypothesised that a greater decline in SpO_2_ following exercise at an early stage of ascent would be seen in those individuals who developed AMS.

## 2 METHODS

### 2.1 Study participants

Participants were part of three separate research expeditions that took place over a 6‐year period: Caudwell Xtreme Everest (2007) (Levett et al., 2010), Caudwell Xtreme Everest 2009 (2009) (Holloway et al., 2014) and Xtreme Everest 2 (2013) (Gilbert‐Kawai et al., 2015). They were aged 18 years or above, permanently resident at low altitude (<1000 m) and were required to pass a two‐stage health screen by experienced expedition doctors. Ethical approval was granted by the University College London Research Ethics Committee and written informed consent was obtained from all participants.

### 2.2 Study setting

**FIGURE 1:**
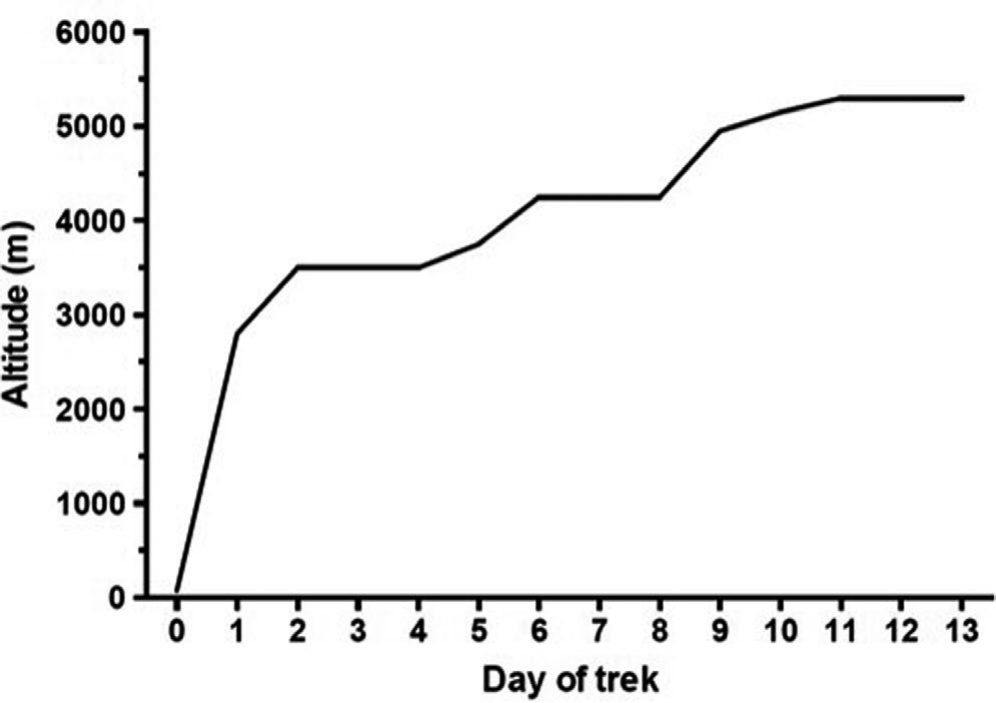
The planned ascent profile for all participants. Daily ascent profile to altitude. In summary, participants went by airplane from Kathmandu (1300 m) to Lukla (2860 m), then ascended on foot. Rest days were inserted into the schedule to allow other experiments to be performed, and to reduce the anticipated incidence of AMS by allowing additional time for acclimatisation (Gilbert‐Kawai et al., 2015; Levett et al., 2010)

Details of the trek to Everest base camp (EBC; 5300 m) have been published previously (Gilbert‐Kawai et al., 2015; Levett et al., 2010). All participants underwent baseline testing, including the Xtreme Everest Step‐Test (XEST), prior to ascent to high altitude, then undertook an 11‐day ascent to EBC and remained there for two further days (Figure 1). All participants followed an identical ascent profile to EBC, with the exception of 14 individuals who deviated for medical or personal reasons. Baseline testing was undertaken in London (75 m); however, 14 participants undertook sea level testing in Kathmandu (1300 m) due to logistical difficulties getting to London. Previous studies have confirmed a similar SpO_2_ between sea level and Kathmandu (Grant et al., 2002). Acetazolamide was not taken prophylactically by any participant, but was administered to those showing symptoms of severe AMS, at the discretion of the expedition medical team.

### 2.3 Physiological measurements and AMS scoring

Each morning participants completed a diary containing the Lake Louise scoring system, and then undertook a short exercise protocol, recording measurements according to written instructions provided to them (Roach et al., 1993). As expeditions took place in 2007, 2009 and 2013, data were collected and analysed using the original Lake Louise scoring system which included the sleep component of the AMS score. The measurements and exercise challenge were conducted at approximately the same time each day, first thing in the morning prior to consuming any food or caffeine. The physiological measurements consisted of resting SpO_2_ and heart rate (HR; Nonin Onyx II 9550, Nonin Medical Inc), respiratory rate (RR) that was counted by a fellow participant and blood pressure (BP) recorded as the mean of three measurements (Omron Intellisense M7, Omron Healthcare Europe B.V.). All resting measurements were taken after participants had been resting (seated) for 5 min (Levett et al., 2010). Following resting measurements, the XEST was undertaken, comprising of 2 min of stepping onto and off a 20 cm high step, at a rate of one step up or down per second, guided by a metronome (Levett et al., 2010). All physiological measurements (with the exception of blood pressure) were repeated during the minute immediately after the XEST was completed (end‐exercise measures). Two Lake Louise score thresholds were used to determine the presence of AMS; 3–4 was considered as mild, while ≥5 was considered moderate to severe. Only scores reaching these thresholds in the presence of a headache were deemed to indicate AMS. No AMS was determined by a score of ≤2 or the absence of a headache regardless of the score.

### 2.4 Data analysis

Data were tested for normality using histograms, and described accordingly. The physiological data used for the primary analysis were from the first morning after arrival at Namche Bazaar (3500 m), as a steep ascent (from 2800 m to 3500 m in 1‐day) is required to reach this altitude. This ascent occurs early in the trek (day 2) and Namche Bazaar is often the first location at which AMS occurs. Independent t‐tests and chi‐squared tests were used for continuous and categorical variables respectively. Occasionally participants were unable to undertake the diary test due to illness or data were incomplete. On these occasions, physiological data from the following day at 3500 m (trek day 3) were used instead (*n* = 9). Sensitivity analyses were undertaken to test the robustness of the results when these participants were removed, and it demonstrated no significant impact on results.

A univariate logistic regression analysis was performed for each physiological and demographic variable. Variables with *p* < 0.15 were included in a multiple logistic regression analysis for moderate‐to‐severe AMS. A ROC curve was performed on the predicted probabilities from the logistic regression to assess the goodness of fit.

All data were analysed using IBM SPSS Version 24 (IBM Corp. released 2013. IBM SPSS Statistics for Windows, Version 23. IBM Corp.). Statistical significance was taken as *p* < 0.05.

## 3 RESULTS

### 3.1 Participant characteristics

**FIGURE 2:**
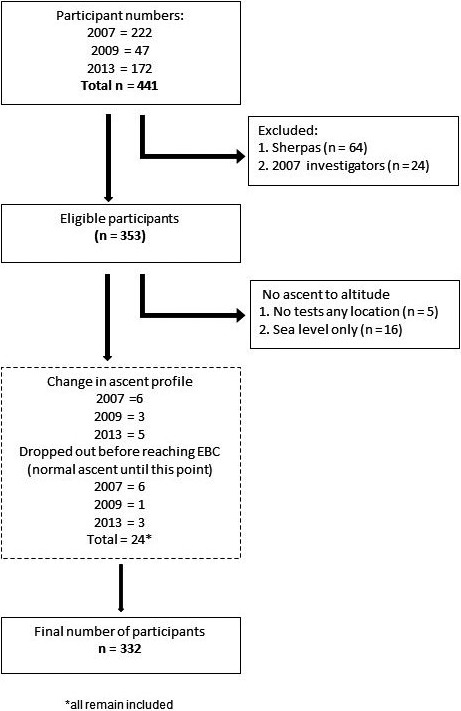
Flow diagram detailing the number of participants included in the final analysis. EBC, Everest Base Camp

The total number of participants across the three expeditions was 441, with 332 eligible for inclusion in this study (Figure 2). A total of 64 participants were excluded from this analysis because they were high‐altitude residents (Sherpa) and 24 because they were Xtreme Everest investigators who followed an alternative (slightly slower) ascent profile. Fourteen participants had a slightly different ascent profile from the main cohort due to illness or personal circumstances, and this resulted in them spending additional days at a lower altitude. Of these fourteen, six had AMS, two had diarrhoea and vomiting, two had respiratory tract infections, one stayed behind with an unwell relative and in three instances the reason could not be determined.

In the study cohort, 56.9% of participants were men and the mean age of participants was 43.1 years (Table 1). The percentage of participants who had previously been exposed to high altitude (>3500 m) was 48.5% (*n* = 160), and of those 6.1% (*n* = 20) self‐reported that they had previously experienced AMS.

**TABLE 1:** Demographic characteristics of participants

Total *n* = 332		Male *n* = 189 (56.9%)	Female *n* = 143 (43.1%)	Total *n* = 332
Age	Mean (95% CI)	44.1 (42.1–46.2)	41.8 (39.6–44.0)	43.1 (41.6–44.6)
Height (cm)	Mean (95% CI)	177.3 (176.2–178.5)	165.6 (164.5–166.7)	172.3 (171.3–173.3)
Weight (Kg)	Mean (95% CI)	80.8 (79.0–82.6)	65.3 (63.7–66.9)	74.1 (72.6–75.6)
BMI	Mean (95% CI)	25.7 (25.2–26.1)	23.8 (23.3–24.3)	24.9 (24.5–25.2)
Previous altitude 3500–5000 m	*n* (% yes)	44 (23.4%)	40 (28.2%)	84 (25.5%)
Previous altitude >5000 m	*n* (% yes)	49 (26.1%)	27 (19.0%)	76 (23.0%)
Previous AMS	*n* (% yes)	9 (4.8%)	11 (7.8%)	20 (6.1%)

### 3.2 Acute mountain sickness

**FIGURE 3:**
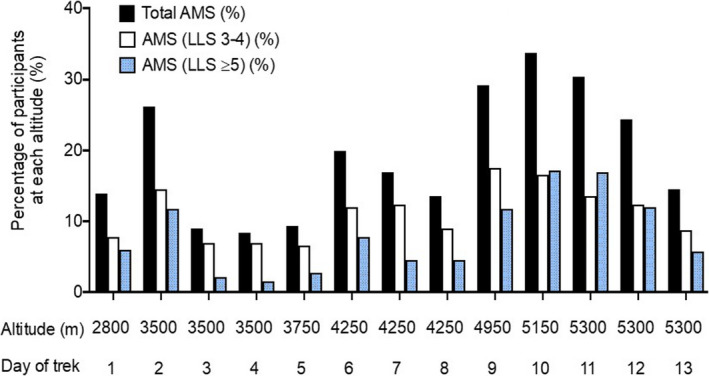
Incidence of acute mountain sickness by trek day. LLS, Lake Louise score

Overall, 244 of 332 participants developed AMS (Lake Louise score of ≥3 with presence of headache) at any point during the expedition (73.5%, 95% confidence interval (CI), 68.7–78.2%). A total of 77 developed mild AMS (23.2%, CI 18.6–27.8%) and 167 developed moderate‐to‐severe AMS (50.3%, 95% CI, 44.9–55.7%). The number of participants with AMS at each day during the trek is shown in Figure 3. There was no difference in gender, age, previous AMS, weight or BMI between participants who developed AMS and those who did not (Table 2). Participants who developed mild AMS were taller than those who did not (174.3 vs. 170.8 cm, *p* = 0.018). The overall incidence of acetazolamide use for treatment of AMS was 19.0% (*n* = 63). Participants requiring acetazolamide for treatment of AMS were older than those who did not require this medication (47.0 years vs. 42.2 years, *p* = 0.015). Participants who had not previously ascended >5000 m were more likely to get moderate‐to‐severe AMS (*p* = 0.006).

**TABLE 2:** Characteristics of participants according to the presence of AMS at any point in the trek and acetazolamide use

Total *n* = 332	No AMS *n* = 88 (26.5%)	AMS (LLS 3–4) *n* = 77 (23.2%)	AMS (LLS ≥5) *n* = 167 (50.3%)	No ACZ *n* = 269 (81.0%)	ACZ *n* = 63 (19.0%)
Age	44.5 (41.1–47.9)	40.7 (37.8–43.6)	43.5 (41.5–45.6)	42.2 (40.5–43.9)	47.0* (43.6 – 50.3)
Height (cm)	170.8 (168.8–172.8)	174.3* (172.2–176.5)	172.1 (170.7–173.5)	172.0 (170.8–173.1)	173.5 (171.3 – 175.7)
Weight (kg)	72.1 (69.3–74.9)	75.9 (72.9–79.0)	74.4 (72.2–76.5)	73.5 (71.9–75.2)	76.7 (73.2 – 80.3)
BMI	24.6 (23.9–25.3)	24.9 (24.1–25.7)	25.0 (24.5–25.5)	24.8 (24.3–25.2)	25.4 (24.5 – 26.3)
Gender		*χ* ^2^ = 1.465, *p* = 0.226	*χ* ^2^ = 0.074, *p* = 0.785	*χ* ^2^ = 0.103, *p* = 0.748	
Previous altitude 3500–5000 m		*χ* ^2^ = 0.158, *p* = 0.691	*χ* ^2^ = 0.001, *p* = 0.981	*χ* ^2^ = 2.155, *p* = 0.142	
Previous altitude >5000 m		*χ* ^2^ = 0.224, *p* = 0.621	*χ* ^2^ = 7.507, *p* = 0.006*	*χ* ^2^ = 0.975, *p* = 0.323	
Previous AMS		*χ* ^2^ = 1.581, *p* = 0.209	*χ* ^2^ = 0.012, *p* = 0.912	*χ* ^2^ = 0.212, *p* = 0.646	

Note

Independent *t*‐tests for continuous (mean, 95% confidence interval) and chi‐squared (*χ* ^2^, *p*‐value) for categorical variables. LLS, Lake Louise score; ACZ, acetazolamide use at some point during the trek.

* *p* ≤ 0.05.

### 3.3 Physiological data

**FIGURE 4:**
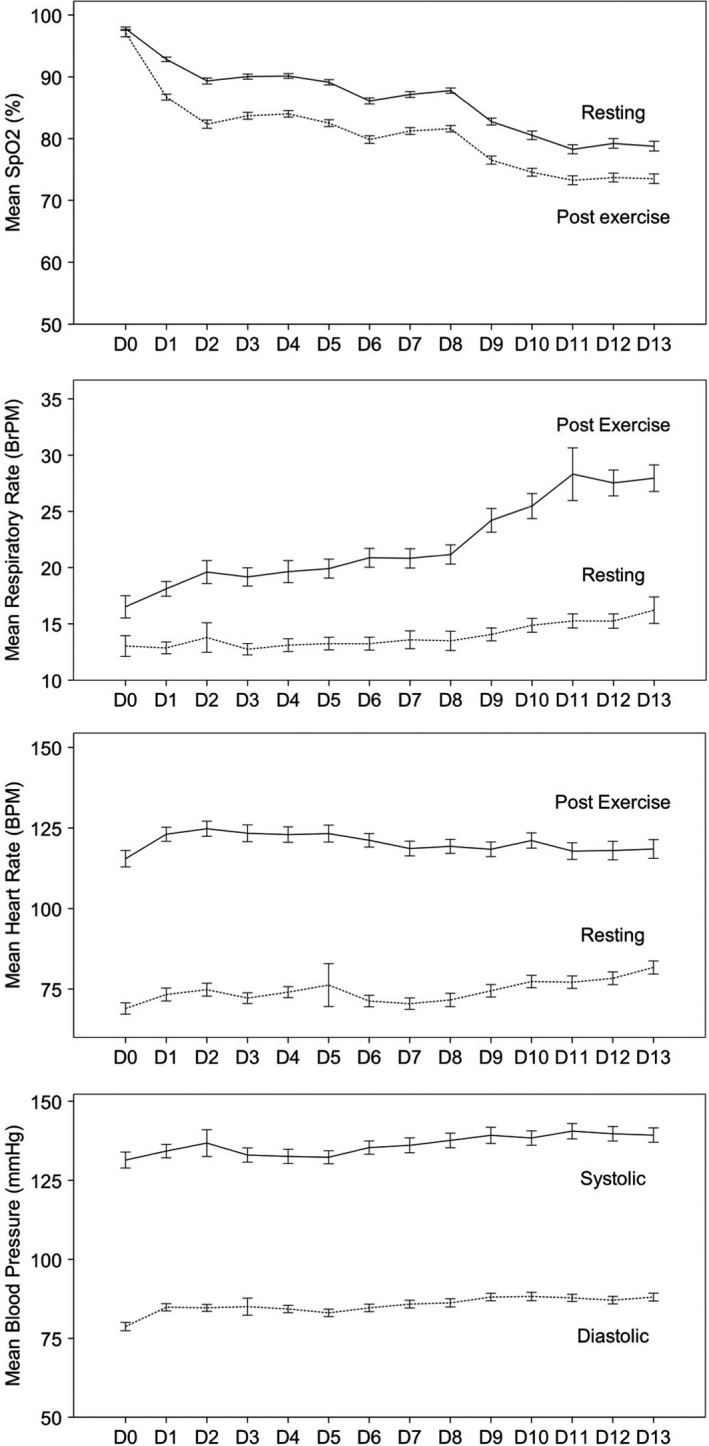
Overall mean (±SEM) change in measured physiological variables in all participants during ascent to high altitude. Solid line = at rest, pre‐exercise; dotted line = at end of exercise. Blood pressure was only measured at rest, prior to exercise

Changes in measured physiological variables on each day of the trek are shown in Figure 4. SpO_2_ decreased both at rest and following exercise as altitude was gained. There was an increase in resting HR, resting RR and end‐exercise RR throughout the ascent, along with an initial increase followed by a decrease in end‐exercise HR. There was a small rise in systolic and diastolic BP throughout the trek.

### 3.4 AMS and physiological measures at concurrent altitudes

Physiological data collected at 3500 m and 5300 m are presented in Tables 3 and 4, respectively, according to participants’ AMS categorisation. At 3500 m, participants who developed mild AMS had lower end‐exercise SpO_2_ (81.1 vs. 82.8%, *p* = 0.016) than those who did not, while those who developed moderate‐to‐severe AMS had a lower resting SpO_2_ (87.6 vs. 89.4%, *p* = 0.006) and lower end‐exercise SpO_2_ (80.7 vs. 82.8%, *p* = 0.007; Table 3). Resting heart rate was also higher in those with moderate‐to‐severe AMS (79.0 vs. 73.4 beats per minute, *p* = 0.015) at 3500 m than those with no AMS. At 5300 m, participants with mild AMS had a lower resting and end‐exercise SpO_2_ than those who did not (76.0 vs. 79.0%, *p* = 0.005 and 71.3 vs. 73.8%, *p* = 0.003 respectively). Similarly, those with moderate‐to‐severe AMS also had a lower resting and end‐exercise SpO_2_ than those who did not (77.3 vs. 79.0%, *p* = 0.022 and 71.7 vs. 73.8%, *p* = 0.007 respectively; Table 4).

**TABLE 3:** Physiological and AMS data from 3500 m (Namche Bazaar)

Total *n* = 329	No AMS at 3500 m *n* = 241 (73.3%)	AMS (LLS 3–4) at 3500 m *n* = 49 (26.7%)	AMS (LLS ≥5) at 3500 m *n* = 39 (11.9%)
SpO_2_ resting (%)	89.4 (88.9–89.8)	88.8 (87.8–89.7)	87.6* (86.4–88.8)
SpO_2_ exercise (%)	82.8 (82.2–83.3)	81.1* (79.9–82.3)	80.7* (79.4–82.0)
SpO_2_ delta (%)	−6.6 (6.1–7.1)	−7.6 (6.4–8.8)	−7.0 (5.7–8.2)
HR resting (bpm)	73.4 (71.7–75.1)	75.8 (72.2–79.5)	79.0* (74.8–83.1)
HR exercise (bpm)	125.1 (123.0–127.1)	123.8 (118.5–129.2)	130.1 (124.5–135.8)
HR delta (bpm)	51.7 (49.4–53.9)	48.0 (41.8–54.2)	51.2 (47.1–55.2)
RR resting (bpm)	13.5 (13.0–14.0)	12.9 (12.0–13.9)	13.9 (12.7–15.1)
RR exercise (bpm)	20.2 (19.4–20.9)	19.6 (18.1–21.1)	20.9 (19.4–22.4)
RR delta (bpm)	6.7 (6.0–7.3)	6.7 (5.6–7.8)	6.8 (5.4–8.3)
BP systolic (mmHg)	133.5 (131.3–135.6)	132.4 (128.2–136.5)	134.7 (129.9–139.4)
BP diastolic (mmHg)	83.7 (82.7–84.8)	83.2 (80.6–85.9)	83.7 (81.0–86.5)

Note

Delta signifies the difference between resting and exercise values. Independent t‐tests for continuous (mean, 95% confidence interval) variables.

* *p* ≤ 0.05.

**TABLE 4:** Physiological and AMS data from 5300 m (Everest Base Camp)

Total *n* = 319	No AMS at 5300 m *n* = 217 (68.0%)	AMS (LLS 3–4) at 5300 m *n* = 45 (14.1%)	AMS (LLS ≥5) at 5300 m *n* = 57 (17.9%)
SpO_2_ resting (%)	79.0 (78.4–79.7)	76.0* (74.1–78.0)	77.3* (75.9–78.7)
SpO_2_ exercise (%)	73.8 (73.1–74.5)	71.3* (69.8–72.9)	71.7* (70.3–73.1)
SpO_2_ delta (%)	−5.2 (4.6–5.8)	−4.6 (2.9–6.3)	−5.6 (4.1–7.1)
HR resting (bpm)	77.4 (75.4–79.3)	78.0 (73.8–82.3)	79.0 (75.5–82.6)
HR exercise (bpm)	119.2 (116.3–122.2)	120.2 (115.2–125.2)	116.1 (110.6–121.6)
HR delta (bpm)	42.2 (39.2–45.1)	42.1 (36.2–48.0)	36.7 (30.6–42.8)
RR resting (bpm)	15.5 (14.8–16.1)	15.0 (13.9–16.2)	15.9 (14.9–16.9)
RR exercise (bpm)	27.9 (26.8–29.0)	28.3 (26.0–30.7)	32.4 (25.1–39.7)
RR delta (bpm)	12.5 (11.6–13.4)	13.4 (11.5–15.4)	16.5 (9.3–23.8)
BP systolic (mmHg)	139.7 (137.3–142.2)	140.0 (135.5–144.6)	137.7 (133.3–142.1)
BP diastolic (mmHg)	87.3 (86.0–88.5)	87.9 (84.9–90.9)	86.4 (84.2–88.7)

Note

Delta signifies the difference between resting and exercise values. Independent *t*‐tests for continuous (mean, 95% confidence interval) variables.

* *p* ≤ 0.05.

### 3.5 AMS and physiological measures from 3500 m

Participants who suffered moderate‐to‐severe AMS at any point during the trek had a lower resting SpO_2_ at 3500 m; 88.5 versus 89.6% (*p* = 0.02; Table 5). Similarly, those who suffered mild or moderate‐to‐severe AMS during the trek had lower end‐exercise SpO_2_ at 3500 m; 82.2 versus 83.8% (*p* = 0.027) and 81.5 versus 83.8% (*p* < 0.001) respectively. Also, participants who experienced mild AMS had lower end‐exercise RR at 3500 m (19.2 vs. 21.3 breaths per minute, *p* = 0.017), in keeping with a reduced hypoxic ventilatory response, consequent low SpO_2_ and an increase in delta SpO_2_. Participants who went on to require acetazolamide had a higher resting RR at 3500 m (14.4 vs. 13.3 breaths per minute, *p* = 0.047).

**TABLE 5:** Physiological data at 3500 m according to AMS at any point during the trek and ACZ use at any point in the trek

Total *n* = 332	No AMS *n* = 88 (26.5%)	AMS (LLS 3–4) *n* = 77 (23.2%)	AMS (LLS ≥5) *n* = 167 (50.3%)	No ACZ *n* = 269 (81.0%)	ACZ *n* = 63 (19.0%)
SpO_2_ resting	89.6 (89.0–90.3)	89.5 (88.6–90.4)	88.5* (88.0–89.1)	89.2 (88.7–89.6)	88.4 (87.5–89.3)
SpO_2_ exercise	83.8 (82.8–84.9)	82.2* (81.2–83.2)	81.5* (80.9–82.1)	82.4 (81.9–82.9)	81.9 (80.8–83.0)
SpO_2_ delta	−5.8 (4.9–6.7)	−7.3* (6.2–8.3)	−7.1* (6.5–7.7)	−6.8 (6.3–7.3)	−6.6 (5.5–7.6)
HR resting	76.1 (73.2–79.1)	74.0 (71.1–76.9)	73.8 (71.8–75.8)	74.9 (73.3–76.5)	72.6 (69.4–75.7)
HR exercise	125.0 (121.3–128.7)	124.0 (120.4–127.7)	126.5 (124.0–129.0)	125.6 (123.6–127.5)	125.3 (120.9–129.6)
HR delta	48.9 (44.7–53.0)	50.1 (46.1–54.0)	52.7 (50.1–55.3)	50.7 (48.6–52.9)	52.4 (48.0–56.8)
RR resting	14.2 (13.3–15.1)	13.0 (12.1–13.9)	13.3 (12.8–13.9)	13.3 (12.8–13.7)	14.4* (13.3–15.4)
RR exercise	21.3 (20.1–22.5)	19.2* (18.1–20.4)	20.0 (19.2–20.8)	19.9 (19.3–20.6)	21.2 (19.7–22.6)
RR delta	7.1 (6.2–8.0)	6.2 (5.4–7.1)	6.6 (5.9–7.4)	6.7 (6.1–7.2)	6.7 (5.3–8.2)
BP systolic	136.4 (132.3–140.5)	132.8 (129.3–136.3)	132.3 (130.0–134.6)	133.8 (131.8–136.1)	132.4 (128.7–136.1)
BP diastolic	83.7 (81.9–85.6)	84.6 (82.5–86.8)	83.2 (82.0–84.4)	83.7 (82.6–84.7)	83.7 (81.7–85.7)

Note

Delta signifies the difference between resting and exercise values. AMS (Lake Louise score 3–4 or ≥5) which occurred at any point throughout the trek. Independent t‐tests (mean (95% confidence interval)). ACZ, acetazolamide use at some point during the trek.

* *p* ≤ 0.05.

### 3.6 Logistic regression analysis

A logistic regression analysis model was conducted to predict moderate‐to‐severe AMS for participants ascending to high altitude. A univariate regression analysis was undertaken for each physiological variable at 3500 m and demographic variables for moderate‐to‐severe AMS. Variables with a univariate significance *p* < 0.15 were then included in a multiple logistic regression analysis.

**FIGURE 5:**
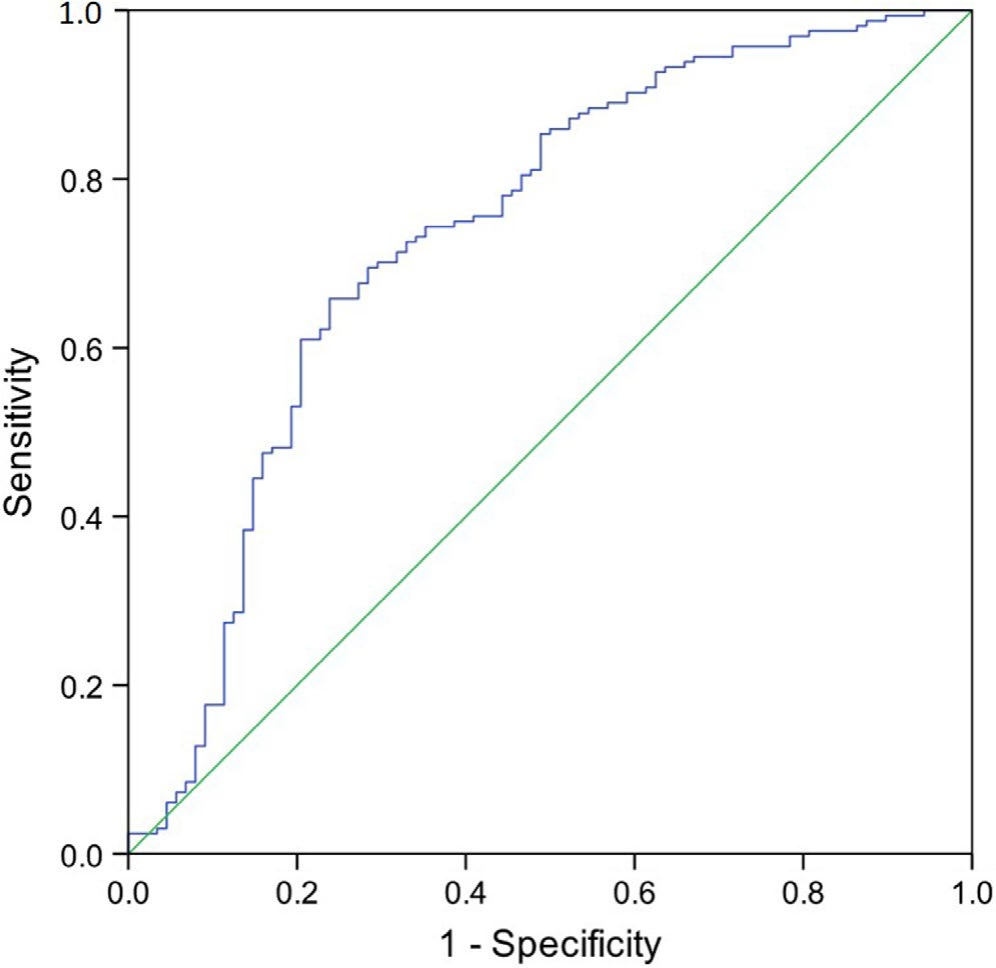
Receiver operator characteristics curve for the moderate to severe AMS logistic regression model. Independent variables included: Resting SpO_2_, end‐exercise SpO_2_, resting respiratory rate, end‐exercise respiratory rate, systolic blood pressure, delta heart rate and no previous altitude exposure >5000 m. Area under the curve = 0.735 (95% CI 0.667–0.804, *p* < 0.001)

The variables included were resting SpO_2,_ end‐exercise SpO_2_, resting RR, end‐exercise RR, delta HR, systolic blood pressure and no previous altitude exposure >5000 m. Despite delta SpO_2_ being a significant variable in the univariate analysis, it was excluded in the multivariate analysis due to the prior inclusion of both resting and end‐exercise SpO_2_ to avoid perfect multicollinearity. The prediction success was 72.6% and the included variables improved the model from 65.1% predicted successfully from the constant alone. End‐exercise SpO_2_ and no previous exposure to altitude >5000 m contributed significantly to the model (*p*‐values <0.001 and 0.003, OR 0.870 and 2.740 respectively; Table 6). The ROC curve for the moderate‐to‐severe AMS logistic regression model had an area under the curve of 0.735 (95% CI 0.667–0.804, *p* < 0.001) confirming the strong predictive value of the model (Figure 5).

**TABLE 6:** Logistic regression analysis for physiological data at 3500 m and prediction of AMS throughout the trek

Predictor (variables with *p* < 0.15 in univariate Logistic Regression analysis)	AMS (LLS ≥5) Odds ratio (95% CI)
Resting SpO_2_	0.963 (0.880–1.055)
Exercising SpO_2_	0.870* (0.803–0.943)
Resting respiratory rate	0.955 (0.877–1.041)
Exercising respiratory rate	0.970 (0.912–1.030)
Systolic blood pressure	0.988 (0.971–1.006)
Delta SpO_2_	Not provided
Delta heart rate	1.016 (1.000–1.033)
No previous altitude exposure >5000 m	2.740* (1.395–5.384)

* *p* ≤ 0.05.

## 4 DISCUSSION

We have described the changes in daily physiological measures in a large cohort of adult volunteers ascending on an identical ascent profile to 5300 m over a period of 11 days. Along with this, we have demonstrated the potential value of a short step‐exercise test conducted at moderate high altitude in predicting the development of AMS.

In keeping with the expected physiological changes with ascent to altitude, we observed a decrease in SpO_2_ and an increase in respiratory rate (both at rest and following exercise) throughout the ascent. This was accompanied by an increase in resting HR and an initial increase in post‐exercise HR, followed by a decrease as further altitude was gained.

A high proportion of participants (73.5%) suffered AMS during the trek using the original Lake Louise criteria and a threshold of ≥3 with a headache. This is similar to the reported incidence of AMS on Kilimanjaro (5895 m; Karinen et al., 2008) but higher than previously reported in the same region of Nepal (Hackett et al., 1976). This is surprising given the relatively slow ascent profile in this study (11 days to EBC), which is more conservative than many EBC trek profiles (Hackett et al., 1976). In our study, the Lake Louise score was recorded daily, in the presence of investigators and a medical team, potentially increasing the likelihood of detecting AMS when compared to other study designs. In previous studies in Nepal, the AMS scores have been recorded less frequently which may have underestimated the incidence of AMS (Hackett et al., 1976). Furthermore, previous studies reporting the incidence of AMS in Nepal have either been cross‐sectional (i.e. recording the incidence at a specific altitude; Basnyat et al., 1999) or did not control the subject ascent profile (Richalet et al., 2012). The ascent profile in our study was tightly controlled and thus the AMS rates reflected variability in individual susceptibility to AMS rather than different rates of hypoxic exposure. Intercurrent illnesses or the impact of other studies being conducted during the Xtreme Everest expedition (e.g. maximal cardio‐pulmonary exercise testing) may have contributed to the high incidence of AMS. Mild AMS occurred in 23.2% of participants versus moderate–severe AMS in 50.3%, suggesting significant pathophysiology in the majority of those developing AMS.

For a number of reasons, we chose to report the findings of this study according to the original Lake Louise criteria (Roach et al., 1993), rather than the revised 2018 criteria (Roach, 2018). Ethical committee approval was for the use of the original criteria and each of the expedition's medical teams used this framework for the detection and treatment of AMS. Furthermore, this was the standard criteria at the time of data collection and using it allows this large dataset to be compared to important published work in this field. It is likely that future research expeditions to high altitude will need to adopt the 2018 criteria.

There was no difference in the incidence of AMS between participants stratified by gender, age, weight or BMI. This is in contrast to previous studies that have reported that being young, female and overweight are risk factors for AMS (Honigman et al., 1993; Richalet et al., 2012; Ri‐Li et al., 2003). Individuals who developed mild AMS were markedly taller (174.3 vs. 170.8 cm), which is difficult to explain from a physiological perspective.

Previous studies report that a history of AMS on a preceding sojourn to high altitude is a risk factor for subsequent AMS (Honigman et al., 1993; Richalet et al., 2012; Schneider & Bernasch, 2002). We found no relationship between previous AMS and AMS on this expedition. We did, however, identify that a previous successful ascent over 5000 m was associated with a lower incidence of AMS. Previous altitude exposure at lesser altitudes (>3500 m but <5000 m) was not associated with lower AMS rates. This may suggest that prior exposure to very high altitude is protective against subsequent AMS, possibly through mechanisms such as epigenetic modification. Alternatively, this may reflect the fact that trekkers who have low susceptibility to AMS (previous successful high‐altitude ascents) are more likely to return to altitude.

The carbonic anhydrase inhibitor acetazolamide is effective for the prophylaxis of and treatment of AMS (Leaf & Goldfarb, 2007). In this study, treatment for AMS was determined by the expedition medical team. All subjects were encouraged to report symptoms of AMS to their medical officer. The treatment algorithm for AMS relied on rest and symptomatic treatment for headache (paracetamol and ibuprofen) in the first instance and escalation to acetazolamide if symptoms did not resolve or worsened. Treatment with acetazolamide therefore relied on self‐reporting of problematic symptoms and not Lake Louise Score alone. Only 19% of participants required treatment with acetazolamide, despite 53% self‐reporting moderate‐to‐severe AMS symptoms on the Lake Louise Score. Participants who were treated with acetazolamide were significantly older than those who were not (47.0 vs. 42.2 years respectively). This may reflect an increase in severity in older participants or reduced symptom tolerance.

### 4.1 The Xtreme Everest step‐test

In addition to resting physiological measurements, participants underwent a daily standardised step‐test exercise challenge. We reported data from 3500 m (day 2) to 5300 m (day 11) to explore relationships between physiological variables during the exercise challenge and AMS incidence and to identify whether the response to the exercise challenge at 3500 m could predict AMS during the ascent.

In keeping with previous studies, we found that participants who developed mild or moderate‐to‐severe AMS had significantly lower resting and end‐exercise SpO_2_ (Burtscher et al., 2004; Faulhaber et al., 2014; Guo et al., 2014; Karinen et al., 2010; Mandolesi et al., 2014). Subjects with mild AMS also had significantly lower end‐exercise RR, which may reflect a reduced hypoxic ventilatory response. A reduced hypoxic ventilatory response and low post‐exercise SpO_2_ have previously been reported to predict AMS risk (Richalet et al., 2012). Richalet and colleagues, in a large prospective cohort study, reported that greater desaturation and low ventilatory response during exercise in an acute normobaric hypoxic challenge at sea level were independent predictors of subsequent severe high‐altitude illness (Richalet et al., 2012). Participants in this study were tested at sea level and then undertook independent excursions to a minimum altitude of >4000 m for three or more days, with a minimum overnight sleeping altitude of >3500 m. A questionnaire was used by subjects to self‐report their ascent profile and physical symptoms of severe high‐altitude illness (SHAI) using Hackett's AMS score. The study had a number of limitations including the use of unaccompanied ascents to altitude, a low response rate (33.2%) leading to risk of selection bias and self‐evaluation of SHAI symptoms without medical guidance risking classification bias.

The use of delta SpO_2_ (before and after exercise) as a predictive measure of AMS was also supported by Karinen and colleagues (Karinen et al., 2010). They found that desaturation after exercise at 3500 m was greater in participants who subsequently developed AMS at 4300 m. However, this relationship was not reproducible at other altitudes (Karinen et al., 2010). Rathat et al. also found, in a retrospective study, that participants who were the most clinically susceptible to AMS had abnormal cardiac and respiratory responses to hypoxia at rest and especially during exercise, thus allowing identification of high‐risk participants (Rathat et al., 1992). This was in agreement with Richalet et al. who found that a ≥22% drop in SpO_2_ after a short exercise test was an independent predictor of severe high‐altitude illness (Richalet et al., 2012). We have demonstrated that a lower SpO_2_ after a standardised exercise test at 3500 m predicted AMS development. The absolute difference in SpO_2_ between those with and without AMS, however, was small (between 1.1% and 2.3% mean absolute difference for resting and post‐exercise SpO_2_ for those with no AMS and with moderate‐to‐severe AMS). Therefore, while the data point towards clear physiological processes that may be associated with the development of AMS, it may be of limited practical utility for screening an individual.

A multivariate regression analysis was undertaken to assess the ability of demographic variables and data from the XEST at 3500 m to predict moderate‐to‐severe AMS at any point during the ascent. The presence of AMS using a threshold of a Lake Louise score of ≥3 was felt to have insufficient specificity (i.e. a high false‐positive rate) for this model. We were able to predict the risk of moderate‐to‐severe AMS using physiological variables from the step test (resting and end exercise SpO_2_, resting and end exercise RR, delta HR and systolic blood pressure) and previous altitude exposure history. This has potential utility in identifying at risk trekkers, who may benefit from a slower subsequent ascent profile, or pharmacological prophylaxis for AMS. Further studies are needed to identify whether performance in the step test in simulated altitude at sea level is of benefit in identifying at risk individuals on a subsequent standardised ascent.

### 4.2 Strengths and limitations

A key strength of this study was the large sample size and tightly controlled ascent profile. The comparison between participants is therefore not confounded by the magnitude or rate of ascent. Furthermore, measurement bias was minimised by blinding all subjects to their measurements and strictly controlling the testing conditions (standardised rest before the test, standardised timing of testing and supervision by trained group leader). Additionally, no prophylactic medications for AMS were taken and acetazolamide use was reserved for those who reported symptoms of AMS to the attendant medical team.

There were, however, a number of limitations to this study. Trekkers were recruited using website and social media adverts that may have led to selection bias. For example, individuals who have previously suffered AMS may be less likely to volunteer to return to altitude. Participants had to self‐finance the expedition which is likely to have limited access to the trek for those in lower socio‐economic groups. Importantly, XEST data from 3500 m were used to predict the development of AMS; an altitude where 26.2% of participants had already developed AMS. Analysis of data from a lower altitude may have mitigated this potential confounding factor. Finally, symptoms were self‐evaluated which may have led to classification bias when determining the Lake Louise score.

## 5 CONCLUSIONS

In a large cohort study of participants undertaking a tightly controlled ascent profile to high altitude, we have demonstrated a higher than previously reported incidence of AMS. Participant characteristics previously shown to predict AMS (gender, age, weight and history of AMS) were not related to the development of AMS in this study. A simple, standardised step test performed at 3500 m was predictive of moderate‐to‐severe AMS during the ascent. Previous extreme altitude exposure (>5000 m) was protective. This simple step test requires further validation both at high altitude and during pre‐trek simulated altitude to evaluate its utility in identifying individuals more likely to develop AMS.

## DECLARATION

Some of the results of this study have previously been reported in a poster and abstract at the 20th International Hypoxia Symposium, Chateau Lake Louise, 2017.

## AUTHOR CONTRIBUTIONS

Substantial contribution to conception or design: D.Z.H.L., K.M., E.G.K., M.G.M., M.P.W.G. and D.S.M. Acquisition of reported data: A.B.C., D.Z.H.L., K.M., E.G.K., P.J.H., M.G.M., M.P.W.G. and D.S.M. Interpretation of reported data: A.B.C., W.A., D.H., P.J.H., M.P.W.G. and D.S.M. All authors were involved in review and critical appraisal of the manuscript and provided approval for submission of the final version.
